# Visualization of the Spiral Ganglion Neuron in Vivo Using a Novel ^177^Lu Nuclear Molecule Label

**DOI:** 10.1002/advs.202504464

**Published:** 2025-05-19

**Authors:** Chenyang Kong, Xiaohui Wang, Jintao Yu, Li Wen, Chengwen Zheng, Ge Yin, Kai Xu, Weiwei He, Hao Wang, Xiaoli Lan, Dawei Jiang, Yu Sun

**Affiliations:** ^1^ Department of Otorhinolaryngology Union Hospital, Tongji Medical College Huazhong University of Science and Technology Wuhan 430022 China; ^2^ Department of Nuclear Medicine Union Hospital, Tongji Medical College Huazhong University of Science and Technology Wuhan 430022 China

**Keywords:** Lu‐177, cochlear implant, hearing loss, Nuclear medicine, spiral ganglion neuron, VGLUT1

## Abstract

For patients with severe and profound hearing loss, cochlear implant (CI), a common and effective modality for restoring hearing, directly stimulates spiral ganglion neurons (SGNs) to generate electrical activity and form auditory perception. However, the postoperative outcome of CI is significantly influenced by the number of surviving SGNs, ​​which is a key focus of preoperative evaluation​​. Existing audiologic function and radiographic tests cannot directly demonstrate the integrity of inner ear primary neurons. In this study, ​​we developed and validated​​ a radionuclide‐labeled anti‐vesicular glutamate transporter 1 (anti‐VGLUT1) antibody‐drug and achieved animal‐level in vivo imaging of cochlear SGNs using nuclear imaging. By screening the public single‐cell sequencing database, it is found that VGLUT1 can serve as a representative cell membrane marker for SGN in the cochlea. The potential of anti‐VGLUT1 conjugated to the long half‐life ^177^Lu as a molecular probe to detect the relative number of SGNs in SGN‐injured mouse and pig models is explored. The study provides a novel method for assessing cochlear nerve integrity in vivo by visualizing target antigen expression levels through nuclear imaging. This approach is promising to help CI candidates with preoperative inner ear SGN integrity assessment, contributing to clinical decision‐making.

## Graphical abstract

The study developed and validated a radionuclide‐labeled anti‐VGLUT1 antibody probe for the first nuclear imaging of cochlear spiral ganglion neurons (SGNs) in vivo. This approach may provide aid in screening candidates suitable for CI surgery by quantifying the number of surviving SGNs, and predicting the potential for postoperative hearing restoration in patients.

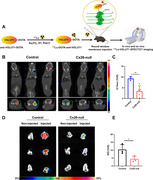

## 1 Introduction

Hearing loss is among the most prevalent sensory impairments, affecting over 5% of the global population, or ≈430 million individuals, with disabling hearing loss.^[^ ^1^ ^]^ By 2050, this number is projected to exceed 700 million, representing one in ten people. To date, over one million individuals worldwide have received cochlear implants (CIs), which are widely recognized as the primary therapeutic option for severe to profound sensorineural hearing loss (SNHL) in both children and adults.^[^ ^2^, ^3^, ^4^, ^5^ ^]^ The outcome of a CI is related to various factors such as the etiology of deafness, cochlear development, integrity of the cochlear nerve and posterior pathway, and speech rehabilitation. Among these factors, the function of the cochlear nerve and posterior pathway is one of the most important factors affecting the outcome of CI, and is also the focus of the preoperative evaluation of CI.

Sound waves are collected by the auricle and travel through the external auditory canal to the eardrum. The eardrum transforms sound waves into movements of the auditory ossicles, which then transmit vibrations to the oval window membrane, inducing vibratory shift of the basilar membrane via endolymph. At this point, the hair cells (HCs) on the organ of Corti (OC) of the basilar membrane convert mechanical energy into electrochemical signals. These signals activate the spiral ganglion neurons (SGNs), generating auditory nerve impulses that travel to the brain via the auditory nerve, where they are processed to produce auditory perception. A variety of factors such as genetics, aging, exposure to ototoxic drugs, and noise can lead to HC death and deafness. Whereas the cochlear implant bypasses impaired sensory HCs and directly stimulates residual SGNs to generate electrical activity and form auditory perception.^[^ ^6^ ^]^ Thus, the number of surviving SGNs is a critical factor influencing CI outcomes.^[^ ^7^, ^8^ ^]^

Currently, preoperative assessments for CI surgery typically include audiological evaluations and imaging studies. These primarily aim to determine whether the patient's hearing loss meets the criteria for CI implantation and, secondarily, whether the anatomical structures are suitable for implantation. However, effectively assessing the number of surviving SGNs in the cochlea remains a significant challenge. Some studies suggest that developing targeted contrast agents could play a pivotal role in identifying the causes and guiding the treatment of hearing loss.^[^ ^9^ ^]^

Immuno‐positron emission tomography (immunoPET) combines highly target‐specific antibodies with radionuclides via coupling agents to generate PET images with high sensitivity and spatial resolution.^[^ ^10^ ^]^ In the era of molecularly targeted therapy and cancer immunotherapy, molecular imaging probes developed based on antibodies, antibody fragments, and nanoantibodies can help to visualize the heterogeneous expression of tumor antigens, assess the pharmacokinetics of therapeutic antibodies in vivo, and predict the efficacy of therapeutic effects, so as to noninvasively and accurately screen patients suitable for treatment.^[^ ^11^ ^]^ PD‐1/PD‐L1, HER‐2, CD4^+^/CD8^+^ targeted radionuclide antibody probes have been clinically translated into molecular imaging, which will provide critical insights​ for clinical treatment.^[^ ^12^, ^13^, ^14^ ^]^ Inspired by the in vivo imaging of these nuclide molecular probes, we attempted to find the target membrane receptor for SGN in the inner ear and visualize the SGNs.

Glutamate (Glu) is the principal excitatory neurotransmitter in the central nervous system (CNS). Within neurons, Glu is transported into synaptic vesicles by vesicular glutamate transporters (VGLUTs).^[^ ^15^, ^16^ ^]^ VGLUTs are key molecules responsible for incorporating glutamate into synaptic vesicles across the nervous system. Among these subtypes, VGLUT1 accounts for the majority of excitatory glutamatergic terminals in the CNS and serves as the most reliable marker for glutamatergic nerve terminals and synapses.^[^ ^17^ ^]^ In the cochlea, inner hair cells (IHCs) are the principal auditory receptors, transmitting sound information to Type I SGNs through glutamate‐mediated ribbon synapses, where numerous small vesicles containing glutamate are tethered.^[^ ^18^, ^19^ ^]^ This study verified the abundant expression of VGLUT1 on the cell membranes of cochlear SGNs.

To address the clinical need for effective preoperative assessment of surviving SGN numbers in cochlear implantation and to leverage the technical advantages of immunoPET, we designed and fabricated a ^177^Lu‐labeled anti‐VGLUT1 antibody for the first time. This study conducted Single‐Photon Emission Computed Tomography (SPECT) imaging of VGLUT1 expression in a mouse model of SGN injury (Sox2CreER Cx26‐null mice) to visualize the number of spiral ganglion neurons. Our results demonstrate that ^177^Lu‐DOTA‐anti‐VGLUT1 is a highly promising radioactive tracer for visualizing the SGNs, owing to its sensitivity, persistence, and target specificity.

## 2 Result

### 2.1 Establishment of a Mouse Model of SGN Injury and Validation with PET Imaging

**Figure 1:**
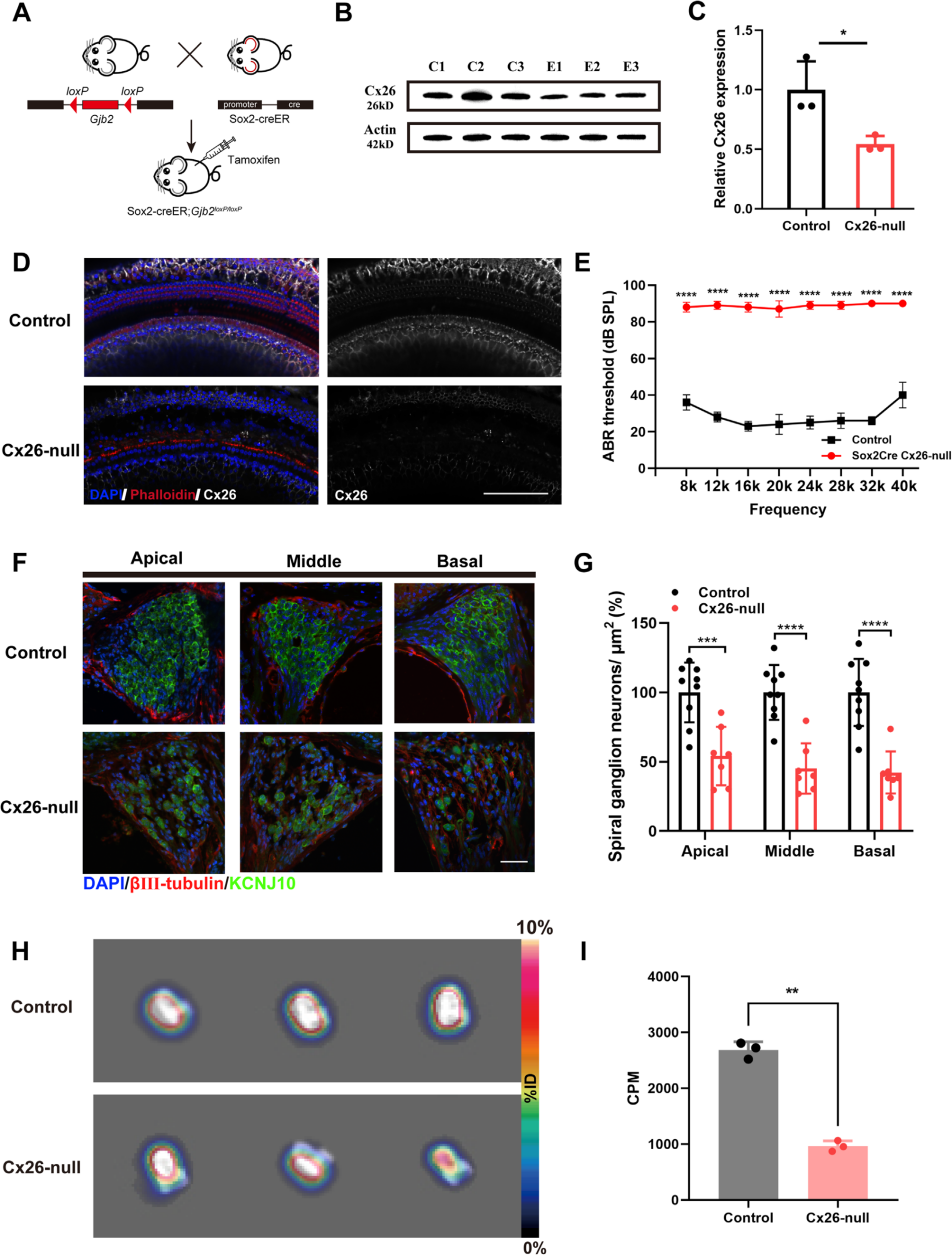
The mouse model of SGN injury was established through targeted knockout of Cx26. A) Schematic representation of the crossbreeding strategy to generate the targeted Cx26‐null mouse model Sox2‐creER; *Gjb2* ^loxp/loxp^. B,C) Western blots showing the decreased protein level of Cx26 in the Cx26‐null mice (E1, E2, E3) compared to the control group (C1, C2, C3). D) Immunofluorescent labeling of the cochlear sensory epithelium for Cx26 (white) in whole‐mount preparations. E) The ABR thresholds of the control and targeted Cx26‐null mice at P30. F) The damage pattern of SGNs in the apical, middle, and basal turn of Cx26‐null mice at 3M. (Tuj1, red) (KCNJ10, green) G) The density of SGNs in the apical, middle, and basal turn was significantly decreased in the Cx26‐null group compared to that in the control group (Control *n* = 8 VS. Cx26‐null *n* = 6). H) ^11^C‐CFT PET imaging of the isolated cochleae of Sox2CreER Cx26‐null mice and littermate negative control mice. (I) The results of Quantitative CPM analysis indicated that the radioactive uptake was lower in the Cx26‐null group (*n* = 3, *p* < 0.01). Error bars indicated the standard deviation. Data are expressed as mean with SEM; ^*^ *p* < 0.05, **p < 0.01, ^***^ *p* < 0.001, ^****^ *p* < 0.0001, significantly different from the control group. SPL, sound pressure level.

Genetic defects, noise exposure, ototoxic drugs, and age‐related deafness can lead to SGN loss.^[^ ^20^, ^21^, ^22^ ^]^ *GJB2* gene mutation is a major causative factor in the CI population, with most of them resulting in severe or profound sensorineural deafness. The Sox2‐iCreERT2; *GJB2* ^loxP/loxP^ mice were subjected to targeted knockout of Connexin 26 (Cx26) in supporting cells (SCs) by tamoxifen‐induced Cre recombinase (**Figure** **1**A). Our previous results indicated that Cx26 in the OC of the mice was significantly deficient throughout the entire length of the basement membrane.^[^ ^23^ ^]^ At first, we verified that Cx26 was successfully eliminated in the cochlea of knockout mice. The results of the western blot confirmed a significant decrease in the Cx26 protein level in the Cx26‐null group (Figure 1B). There was a statistically significant difference between the two groups (*p* < 0.05, Figure 1C). Additionally, immunofluorescence staining of the cochlear basement membrane in 1‐month‐old (1M) Sox2CreER Cx26‐null mice also revealed the absence of Cx26 in the OC region, including DCs and PCs (Figure 1D). Subsequently, Auditory brainstem response (ABR) tests were performed on Cx26‐null and wild‐type group at 1M. The Cx26‐null mice exhibited severe hearing loss over the entire frequency range, in sharp contrast to the control group (*p* < 0.0001, Figure 1E). The hearing thresholds in the Cx26‐null group at 8, 12, 16, 20, 24, 28, 32, and 40 kHz were 88 ± 2.45, 89 ± 2, 88 ± 2.45, 87 ± 4, 89 ± 2 dB, 90, and 90 dB sound pressure level (SPL), respectively (Figure 1E). This result is consistent with our previous studies.

Next, we focused on the damage of the SGNs in Sox2CreER Cx26‐null mice. Our previous studies demonstrated that targeted deletion of Cx26 in Sox2^+^ SCs causes abnormal innervation, demyelination, and degeneration of SGNs. KCNJ10 is the major K^+^ channel in the satellite glial cells surrounding SGNs, and βIII‐tubulin (Tuj1) can specifically label SGN. Immunofluorescence staining for KCNJ10 and Tuj1 was carried out at 3M, and the results showed that both expressions declined along with the degeneration of the SGNs (Figure 1F). Quantitative analysis indicated that the density of SGNs in the apical, middle, and basal turns of the Cx26‐null group decreased by 45.87% (*p* < 0.001), 54.82% (*p* < 0.0001), and 57.73% (*p* < 0.0001), respectively (Figure 1G).

To explore whether the PET/CT molecular imaging technique can visualize the cochlear nerves, we first targeted the dopamine transporters (DATs), which is extensively utilized in the clinical diagnosis of neurodegenerative diseases, to conduct PET/CT imaging on the animal models of SGN injury. For Sox2CreER Cx26‐null mice, PET scans were performed on these mice and their Cx26^loxP/loxP^ littermates by injecting 2 µL of ^11^C‐2β‐Carbomethoxy‐3β‐(4‐fluorophenyl) tropane (^11^C‐CFT) into the cochlea through the round window membrane (RWM). The maximum intensity projection (MIP) images of the isolated cochleae showed that the Cx26‐null group had a lower ^11^C‐CFT uptake than the control group (Figure 1H). We performed quantitative analysis on the cochlea: the ^11^C‐CFT uptake value of the Cx26‐null group was found at 962.9 ± 77.8 counts per minute (CPM) and that of the control was 2450.2 ± 324.4 CPM (*n* = 3, *p* < 0.01) (Figure 1I).

### 2.2 Establishment of Pig Model of SGN Injury and Validation with ^11^C‐CFT PET Imaging

**Figure 2:**
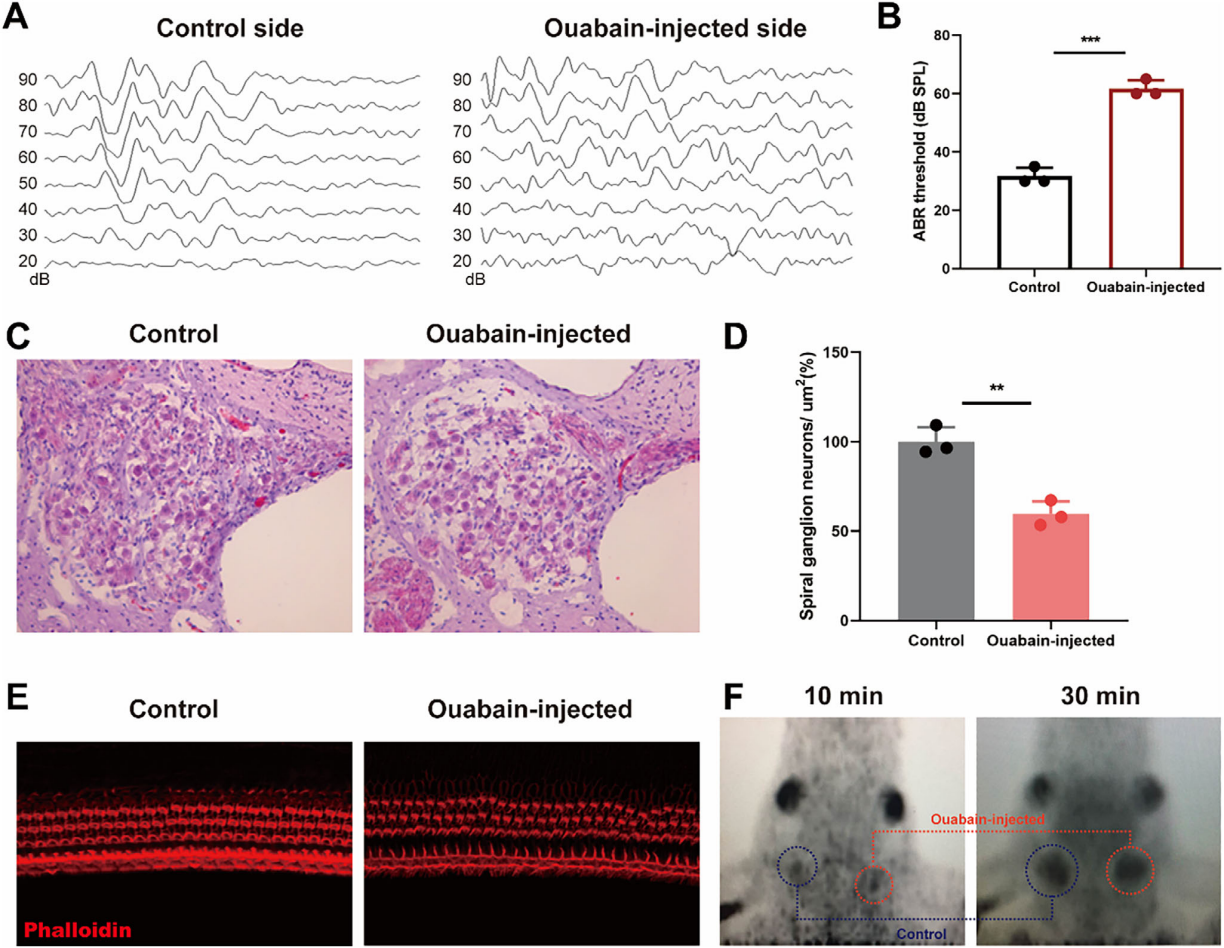
^11^C‐CFT PET Imaging of pig model with SGN injury. A,B) ABR click thresholds in both ears of pig after being administered ouabain for 7 days. C) The results of SGNs loss after Ouabain administration for 7 days. D) The density of SGNs was significantly decreased in the injected group compared to that in the non‐injected group. E) The observation results of cochlear HCs in the ouabain‐injected and control group. F) ^11^C‐CFT PET imaging of the pig 7 days after Ouabain Administration. Data are expressed as mean with SEM (normally distributed); Data analysis was conducted using the unpaired *t*‐test and F‐test demonstrating homogeneity of variance among the groups (*p* > 0.05). ^**^ *p* < 0.01, ^***^ *p* < 0.001, significantly different from the control group.

Minipigs are closer to humans in organ structure and metabolism and share common pathogenic mechanisms with humans in many diseases. It is an ideal animal model for conducting research on a wide range of human diseases. In terms of hearing, since the inner ear structure and hearing of minipigs are also mature at the late embryonic stage, minipigs have an incomparable advantage in the establishment of animal models of human deafness.^[^ ^24^, ^25^ ^]^ The SGNs, spiral rim, HCs, and marginal cells of the vascular stripe in the cochlea express Na^+^‐K^+^‐ATPase, which plays an important role in maintaining the ionic gradient of the endolymphatic and perilymph fluids, as well as in stabilizing the function of each cell. Ouabain, a potent inhibitor of the Na^+^‐K^+^‐ATPase, induced SGN injury in the inner ear of minipigs, and the effect of the injury was concentration‐ and time‐dependent. No significant damage to HCs was observed.^[^ ^26^, ^27^ ^]^ Bama miniature pigs were locally administered 10 mm ouabain through the RWM for 7 days. ABR tests were performed on both ears of the pig 7 days after administration. The experimental side had click thresholds of 60 dB SPL versus 30 dB SPL in control (**Figure** **2**A,B). HE section staining revealed significant impairment of SGN bodies and nerve fibers in the cochlea on the ouabain‐injected side, compared to the non‐injected ear (Figure 2C). Quantitative analysis showed that the SGN density in different segments of the experimental cochlea was significantly lower than that in the untreated cochlea (*n* = 3, *p* < 0.01) (Figure 2D). Meanwhile, no obvious damage to HCs was observed (Figure 2E). PET/CT images showed bilateral SGN tracer uptake differences. The imaging results revealed that the radioactive uptake on the ouabain‐injected side was slightly lower than that of the control side (Figure 2F). However, the imaging resolution of CFT‐PET was not high enough to differentiate the extent of bilateral SGN damage. This suggests the need for further exploration of more specific radiotracers for enhanced imaging.

### 2.3 Screening and Identification of a Novel SGN Specific Membrane Receptor

**Figure 3:**
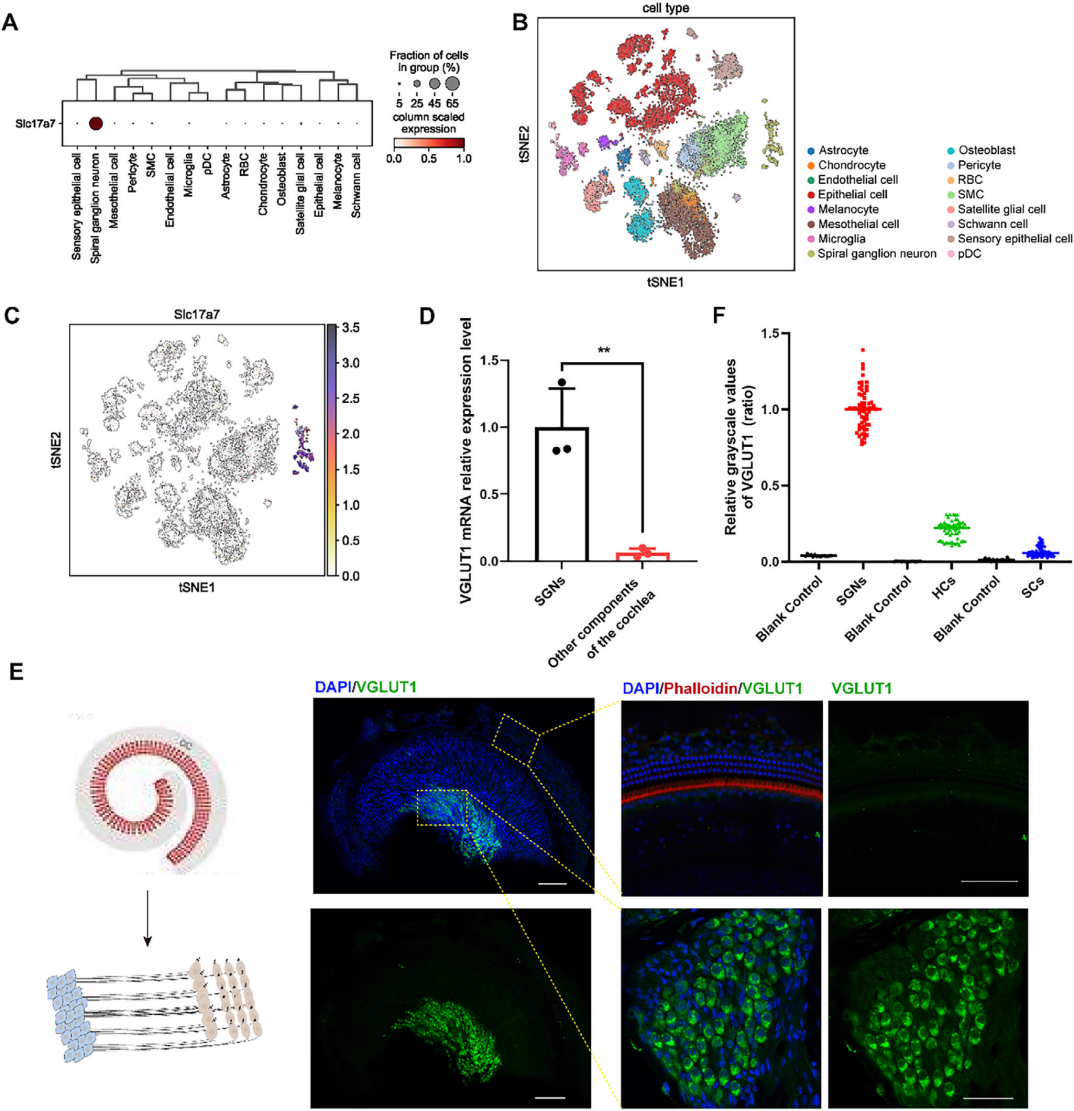
Identification of spiral ganglion‐specific membrane protein VGLUT1. A) The bubble chart shows the expression pattern of *Slc17a7* in various cells of the cochlea. B) tSNE plot of distinct cell types from the cochlea of the C57/BL6 mice at postnatal 28. Sixteen main cell clusters were defined. SGNs in yellow. C) tSNE plot visualizes that *Slc17a7* is highly expressed in SGNs. D) VGLUT1 mRNA relative expression level in the SGNs and other components of the cochlea. E) The immunofluorescence staining in different dimensions shows the expression pattern of VGLUT1 (green) in the cochlea. VGLUT1 was expressed strongly in SGNs but undetectable in the HCs of the cochlea at P30. F) Quantification of immunolabelling of VGLUT1 in the SGNs, HCs, and SCs from the cochlea of wild‐type (15 cells in each type cell from four mice); blank control referred to using only the secondary antibody (Goat anti‐Rabbit, Alexa Fluor 488, Invitrogen) as a nonspecific staining control. Abbreviations: RBC, Red blood cell; SMC, Smooth muscle cell; pDC, Plasmacytoid dendritic cell; SGN, Spiral ganglion neuron; HC, Hair cell; SC, Supporting cell. Data are expressed as mean with SEM (normally distributed). When performing the unpaired *t*‐test, the F‐test analysis revealed homogeneity of variance among the groups (*p* > 0.05). ^**^ *p* < 0.01, significantly different from the control group.

A membrane protein, VGLUT1, encoded by *Slc17a7*, representatively expressed in the spiral ganglion was selected and verified through multiple approaches. First, in the early stage of this study, the single‐cell RNA sequencing data of postnatal 28 (P28) wild‐type mice from Zuo et al. were utilized. After quality control (QC) to filter out low‐quality cells, 19 054 sequencing genes for each biological replicate were obtained. Nearest neighbor unsupervised clustering was employed to identify cell clusters, and the t‐distributed stochastic neighbor embedding (t‐SNE) plot based on the expression patterns was used for visualizing the cell clusters (**Figure** **3**B). After screening, the expression of *Slc17a7* in different cell types was focused on. The bubble chart and t‐SNE results indicated that VGLUT1 was highly expressed in the cochlear SGNs, perhaps being a representative membrane protein of the SGN (Figure 3A,C). Subsequently, the expression pattern of VGLUT1 was verified. The PCR results for the SGNs and the remaining components of the cochlea in 1M wild‐type mice demonstrated that the expression of VGLUT1 in the SGNs was significantly higher than that in other cells of the cochlea (*p* < 0.01, Figure 3D). Fluorescence images of the cochlea in 1M mice also revealed that VGLUT1 was highly expressed in the SGNs and scarcely expressed in other cell types (Figure 3E). Quantitative analysis of SGNs, HCs, and SCs indicated that the expression of VGLUT1 in the cochlear spiral ganglion was significantly higher than that in sensory epithelial cells and SCs (Figure 3F). Based on the specificity of VGLUT1 expression in the SGNs, we contemplated that it might be the targeted antibody requisite for SGN imaging.

### 2.4 Ex Vivo ^177^Lu‐anti‐VGLUT1 SPECT/CT Imaging and Validation of VGLUT1 Degradation of SGN Injury Mice Model

**Figure 4:**
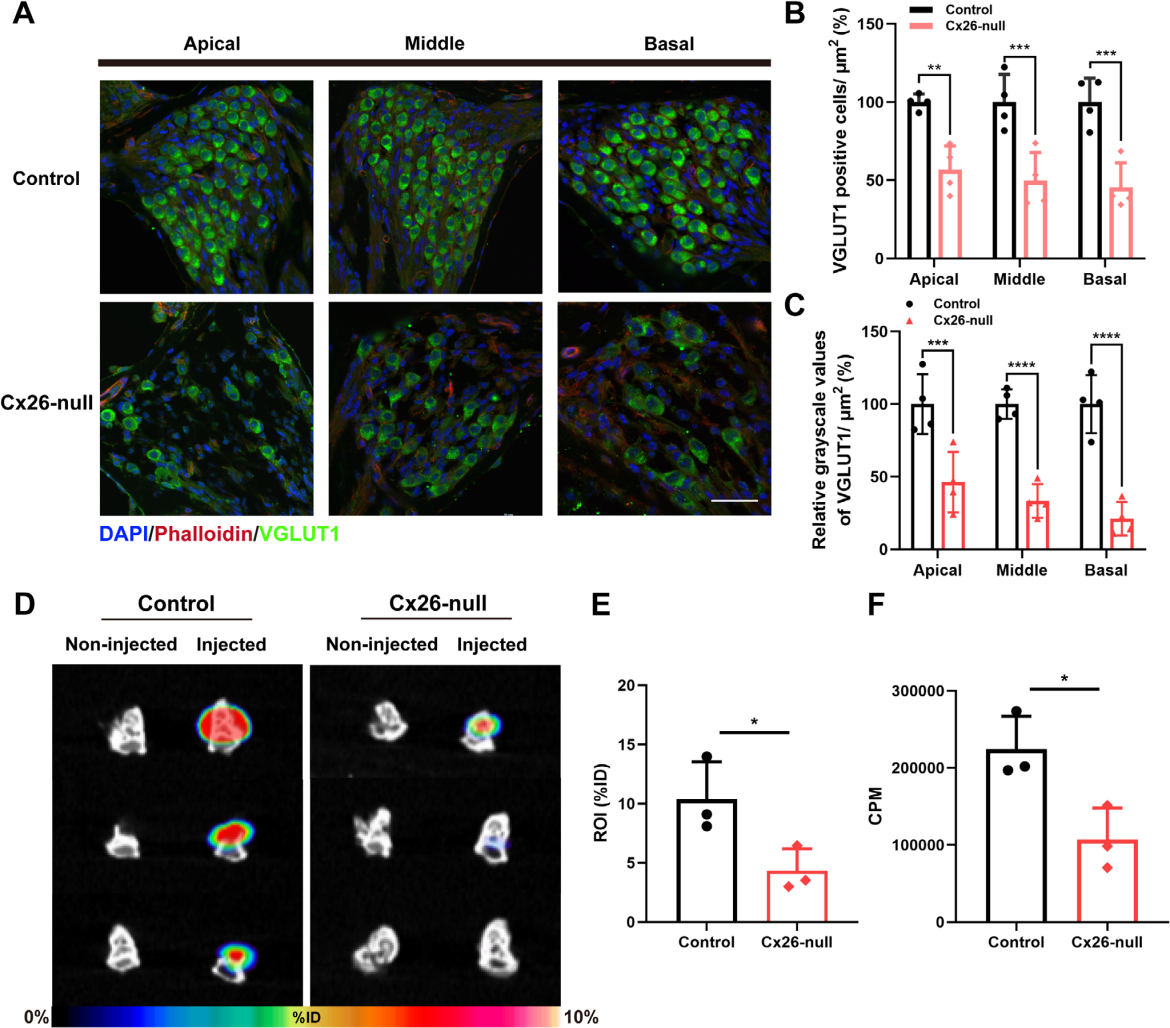
The expression and ex vivo SPECT/CT imaging verification of VGLUT1 in Cx26‐null mice. A) Immunofluorescent staining of VGLUT1 (green) in the apical, middle, and basal turn of SGNs in the control and Cx26‐null groups. Scale bars = 50 µm. B) Quantification of the immunolabeling of VGLUT1 per unit area in the spiral ganglion of the control group and the Cx26‐null group (*n* = 4). C) The number of VGLUT1‐positive cells was significantly lower in the Cx‐26 group than in the control group for apical, middle, and basal turns. D) The ^177^Lu‐anti‐VGLUT1 SPECT/CT images of the injected and noninjected sides of cochlea ex vivo in the Cx26‐null and control groups. E) Quantitative regions of interest (ROI) analysis of the cochlea ex vivo showed lower ^177^Lu‐anti‐VGLUT1 uptake in the Cx26‐null group than in the Control group (*n* = 3, *p* < 0.05). F) Quantitative CPM analysis of the cochlea ex vivo showed lower ^177^Lu‐anti‐VGLUT1 uptake in the Cx26‐null group than in the Control group (*n* = 3, *p* < 0.05). Data are expressed as mean with SEM (normally distributed). When performing the unpaired *t*‐test, the F‐test analysis revealed homogeneity of variance among the groups (*p* > 0.05). ^*^ *p* < 0.05, ^**^ *p* < 0.01, ^***^ *p* < 0.001, ^****^ *p* < 0.0001, significantly different from the control group.

It is known that there is degeneration of the SGNs and neural pathways in Cx26‐null mice. To further ascertain whether the expression of VGLUT1 in Cx26‐null mice also declined, we conducted VGLUT1 immunostaining on the cochlea of Cx26‐null mice. The results indicated that the number of VGLUT1‐positive cells in SGNs decreased in the Cx26‐null (*n* = 4) compared to that in the control group (*n* = 4) (**Figure** **4**A). Quantitative evaluation confirmed this finding: compared with the control group, the number of VGLUT1‐positive cells in apical, middle, and basal turns was significantly decreased in the Cx26‐null group, representing 56.67%, 49.92%, 45.41% of the control group, respectively (two‐way ANOVA, *p *< 0.01, Figure 4B). Meanwhile, immunofluorescence quantification further disclosed that VGLUT1 expression in SGNs of the apical, middle, and basal turns was also significantly reduced compared to the control group, accounting for 46.26%, 33.34%, 21.05% of the control group, respectively (two‐way ANOVA, *p *< 0.001, Figure 4C).

Owing to the suboptimal imaging effect of the ^11^C‐CFT utilized in our previous studies, we developed a more specific, long‐lasting, and accurate radiotracer by combining the SGN‐specific membrane protein VGLUT1 and the long half‐life nuclide ^177^Lu. Following the injection of ^177^Lu‐DOTA‐anti‐VGLUT1 through RWM, the Cx26‐null mice and the control mice were sacrificed for individual ex vivo cochlear SPECT/CT imaging. Ex vivo histology confirmed the low VGLUT1 expression on the cochlea of Cx26‐null mice (*n* = 3, *p* < 0.05). Meanwhile, we found that the noninjected side of the cochlea did not show ^177^Lu‐anti‐VGLUT1 radioactivity (Figure 4D,E). The ^177^Lu‐anti‐VGLUT1 uptake value of the experiment group was 125906 ± 43967 CPM and the control was 224135 ± 35052 CPM (*n* = 3, *p* < 0.05) (Figure 4F). The results indicate that the reduction of VGLUT1 expression in SGN‐injured mice is consistent with the radioactive quantitative results of the cochlea.

### 2.5 ^177^Lu‐Anti‐VGLUT1 Dynamic SPECT/CT Imaging in the SGN Injury Mouse Model

**Figure 5:**
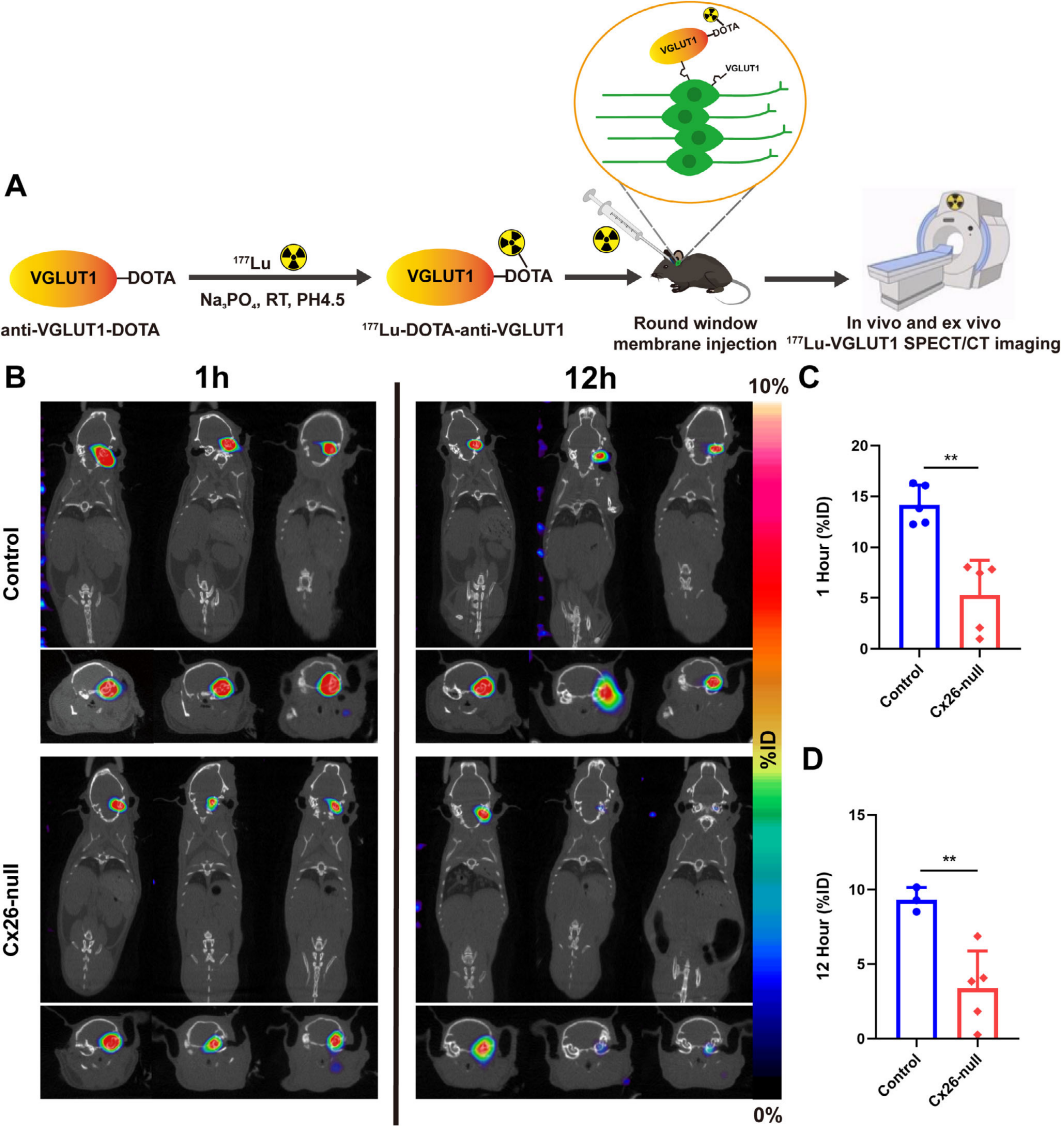
^177^Lu‐anti‐VGLUT1 dynamic SPECT/CT imaging and ROI analysis of Cx26‐null mice. A) Scheme of the experiment. VGLUT1 was first conjugated with DOTA and then labeled with ^177^Lu. It was injected into the cochlea of mice through the RWM, followed by in vivo and ex vivo SPECT imaging. B) ^177^Lu‐anti‐VGLUT1 SPECT/CT images of Cx26‐null group and Control group at 1 and 12 h after injection of ^177^Lu‐DOTA‐anti‐VGLUT1 (*n* = 3). C,D) Quantitative ROI analysis of the cochlea at 1 and 12 h showed a lower ^177^Lu‐anti‐VGLUT1 uptake value in the Cx26‐null group than in the Control group (1 h, *n* = 5, *p* < 0.01; 12 h, Control *n* = 3 vs Cx26‐null *n* = 5, *p* < 0.01). Data are expressed as mean with SEM (normally distributed); Data analysis was conducted using the unpaired *t*‐test and F‐test demonstrating homogeneity of variance among the groups (*p* > 0.05). ^**^ *p* < 0.01, significantly different from the control group.

Small animal dynamic SPECT imaging was performed to demonstrate the effectiveness of ^177^Lu‐DOTA‐anti‐VGLUT1 as a targeted probe for the detection of VGLUT1 expression and SGN imaging in vivo (**Figure** **5**A). The 3M Cx26‐null mice and their Cx26^loxP/loxP^ littermates underwent SPECT imaging at 1 and 12 h after injecting 2 µL of ^177^Lu‐DOTA‐anti‐VGLUT1 through the RWM. The MIP images at two‐time points showed that ^177^Lu‐anti‐VGLUT1 accumulated in the cochlea on the injection side, while no accumulation was detected on the opposite side. Moreover, the Cx26‐null group exhibited a lower ^177^Lu‐anti‐VGLUT1 uptake than the control group (Figure 5B). Quantitative ROI analysis was performed on cochlea (Figure 5C,D); the 1h ^177^Lu‐anti‐VGLUT1 uptake value of the experiment group was 5.27 ± 3.09 %ID and that of the control was 14.18 ± 1.74 %ID (*n* = 5, *p* < 0.01); the 12 h ^177^Lu‐anti‐VGLUT1 uptake value of the experiment group was 3.37 ± 2.23 %ID and that of the control was 9.13 ± 0.67 %ID (Control *n* = 3 vs Cx26‐null *n* = 5, *p* < 0.01). The significant difference between Cx26‐null and the Control group indicated ^177^Lu‐DOTA‐anti‐VGLUT1 specifically bound to VGLUT1. However, we observed the rapid clearance of ^177^Lu ‐DOTA‐anti‐VGLUT1 in the cochlea by comparing the SPECT images at 1 h and 12 h.

## 3 Discussion

Cochlear implants serve as the primary treatment for severe or profound sensorineural hearing loss, and the preservation of cochlear nerve components constitutes a crucial determinant for a favorable prognosis. However, for patients with sensorineural hearing loss, how to assess the subsequent efficacy of the cochlear implant preoperatively poses a long‐standing conundrum for clinicians. In this study, we report that the imaging of the cochlear nerve in animal models of hearing loss can be accomplished in vivo through the utilization of immuno‐PET probe, and that ^177^Lu‐DOTA‐anti‐VGLUT1 can effectively evaluate the expression level of VGLUT1 in the cochlea, further visualizing the relative quantity of SGNs in mice with SGNs loss. Quantifying the remaining number of SGNs can predict the potential for hearing recovery in deafness patients following cochlear implantation, providing a basis for identifying appropriate candidates for CI surgery. These findings highlight the application potential and translational value of immuno‐PET probes in the field of hearing research.

We established appropriate animal models applicable to the research of cochlear nerve imaging. *GJB2* (encoding Cx26) mutations are the most common cause of nonsyndromic hereditary deafness (DFNB1).^[^ ^28^ ^]^ Deafness severity caused by various *GJB2* mutations varies significantly and often coincides with SGN and auditory neural pathway degeneration. Our previous studies revealed that the Sox2Cre; Cx26^loxP/loxP^ knockout mouse model exhibited severe hearing loss and extensive loss of HCs, resembling the manifestations of severe SNHL clinically critical population requiring CI to improve hearing. Furthermore, targeted Cx26 knockout in Sox2^+^ SCs caused multiple and varying degrees of pathological changes in primary auditory neurons and nerves, leading to suboptimal and unstable CI outcomes.^[^ ^23^ ^]^ This study found significant SGNs and nerve damage in Cx26‐null mice at three months, which was manifested as the reduction of the SGN density in the apical, middle, and basal turns. Based on these findings, we selected the 3M Sox2CreER Cx26‐null mouse model for evaluating immunoprotein in in vivo SPECT/CT imaging of cochlear SGNs. Details of the *GJB2* knockout approach have been described in our previous studies.^[^ ^29^, ^30^ ^]^ Simultaneously, we developed a Bama miniature pig model as an ideal platform for inner ear experiments. The left ear served as the experimental ear in this model. Ouabain (10 mm) was locally administered via RWM through an endoscopic trans‐tympanic membrane puncture to damage cochlear SGNs, with the right ear left untreated as the control. Seven days after administration, the experimental ear showed 60 dB SPL hearing loss, with significant SGNs damage.

^11^C‐CFT, a DAT PET imaging agent with high affinity and selectivity for dopamine transporterand resistance to metabolic degradation, is commonly used for brain imaging to diagnose neurodegenerative diseases such as Parkinson's disease (PD), reflecting dopaminergic neuron damage.^[^ ^31^, ^32^, ^33^ ^]^ Dopamine receptors are also widely expressed in cochlear SGNs,^[^ ^34^, ^35^ ^]^ prompting us to test ^11^C‐CFT in the two aforementioned animal models. In preliminary experiments, we attempted several administration methods. Systemic administration, such as intravenous or intramuscular injection, failed to result in radioactive accumulation in the cochlea due to the blood‐labyrinth barrier (BLB); Tympanic cavity administration led to significant radionuclide leakage through the eustachian tube and insufficient cochlear uptake, resulting in poor imaging quality. Thus, we ultimately selected RWM injection in mice as the most direct method for delivering radiotracer. This method allows radionuclides to diffuse from the internal wall of the scala tympani into Rosenthal's canal, minimizing their diffusion to other cochlear regions and enhancing their binding to SGNs. Consequently, it facilitates a more intuitive assessment of the probe's labeling efficiency with SGNs. Ex vivo PET imaging showed lower cochlear uptake in Cx26‐null mice compared to controls. However, the imaging resolution was low, and the SGN labeling effect remained suboptimal. For the miniature pig model with Ouabain‐induced SGN injury, ^11^C‐CFT was injected via the auricular vein, and scanning was performed at 10 min later. No significant difference in cochlear uptake was observed between experimental and control ears, preventing accurate differentiation of bilateral SGN injury. Considering the short physical half‐life (20.4 min) and rapid metabolism of ^11^C, it might not be suitable as a radiotracer for cochlear nerve imaging. Developing a radiotracer with strong targeting ability, slow metabolism, and low toxicity is crucial for characterizing cochlear nerves and improving the diagnosis and treatment of SNHL. At this point, antibody‐based immunoPET tracers capable of targeted labeling have attracted our attention.

ImmunoPET is a groundbreaking molecular imaging technique combining the high targeting specificity of monoclonal antibodies (mAbs) with the sensitivity of PET technology.^[^ ^10^ ^]^ For example, Her2 is commonly expressed on tumor cell membranes. The corresponding antibody, trastuzumab, is radiolabeled in vitro to generate immunoPET probes. Upon injection into the body, these probes bind to the membrane receptors, enabling the visualization of Her2‐positive breast cancer lesions through immunoPET imaging.^[^ ^36^, ^37^ ^]^ This research is currently under the clinical trial stage (NCT05955833).^[^ ^38^ ^]^ A key feature of this method is that the antibody remains extracellular, binding exclusively to membrane surface receptors. VGLUT1, a critical molecule for transporting the excitatory neurotransmitter glutamate into synaptic vesicles in the CNS, is responsible for most excitatory glutamatergic terminals.^[^ ^17^ ^]^ Furness & Lawton reported that VGLUT1 was located in the cytoplasm of IHCs, where it appeared as a small scattered fluorescent puncta, implicating vesicular glutamate release in sound signal transmission.^[^ ^39^ ^]^ In contrast, Seal et al. and Edwards et al. detected no immunoreactivity for VGLUT1 in IHCs/OHCs across all ages using immunofluorescence, RT‐PCR, and gene labeling.^[^ ^40^, ^41^ ^]^ In our study, we have confirmed the high expression of VGLUT1 on the cell membranes of SGNs through single‐cell sequencing analysis, RT‐PCR, and immunofluorescence, as well as its low expression in IHCs, aligning with Furness & Lawton's findings. Semi‐Quantitative immunofluorescence analysis showed that VGLUT1 expression in IHCs was only 20.8% of that in SGNs, significantly lower and insufficient to interfere with SGN radiolabeling. Based on these experimental data and analyses, we identified VGLUT1 as a representative SGN membrane protein marker and developed radionuclide molecular probes targeting it.

Given the small size and deep location of the cochlea, the physical properties of ^177^Lu make it compatible for use in our study. Its low beta‐ray energy minimizes bone marrow suppression, reduces surrounding tissue damage, and concentrates energy effectively within the cochlea. Additionally, the long half‐life of ^177^Lu (6.73 days) makes it more favorable for long time monitoring.^[^ ^12^ ^]^ Based on these characteristics, we employed a highly active ^177^Lu‐labeled anti‐VGLUT1 antibody, which was injected via the RWM into the cochlea to target SGNs. Additionally, by conducting in vitro cell uptake and blocking experiments of SGNs co‐incubated with the probe, we confirmed the probe's s specific and targeted binding to SGNs.

In SPECT/CT imaging, ^177^Lu‐anti‐VGLUT1 radioactivity was confined to the cochlea, with no detectable radioactivity in other parts of the mice. In the control group, cochlear uptake of ^177^Lu‐anti‐VGLUT1 was 14.18 ± 1.74 %ID at 1 h and 9.13 ± 0.67 %ID at 12 h postadministration, significantly higher than in the Cx26‐null group (1 h: 5.27 ± 3.09 %ID; 12 h: 3.37 ± 2.23 %ID). Cochlear uptake progressively declined over time, indicating a stable metabolic process within the cochlea. Moreover, ex vivo cochlear CPM demonstrated a significant difference between the experimental and control groups (a 52.33% decrease). Semi‐quantitative fluorescence analysis of VGLUT1 demonstrated that its expression in SGNs of Cx26‐null mice was, on average, 66.45% lower than that in the control group and significantly higher than its expression in other cochlear components (including IHCs). This suggests that in SPECT/CT imaging, interference signals from VGLUT1 expression in non‐SGN cochlear cells are unlikely to significantly affect overall image results. Moreover, the number of VGLUT1‐positive SGN cells in Cx26‐null mice decreased by ≈49.33%, consistent with ex vivo CPM data, further supporting the reliability of SPECT/CT imaging. Collectively, these findings demonstrate that immunePET imaging can effectively evaluate VGLUT1 expression in the cochlea and indirectly reflect the relative number of SGNs.

Currently, routine preoperative screening methods for CI (such as otoacoustic emissions, cortical evoked potentials, and CT/MRI) are capable of assessing the degree and type of hearing loss in patients, as well as observing the developmental status of the middle ear, inner ear, and auditory nerve, along with detecting potential structural abnormalities.^[^ ^42^ ^]^ However, these methods cannot directly evaluate the morphology and function of SGNs. In previous studies, the small size and deeply embedded location of the cochlea hindered the exploration of nuclear imaging for this organ. Our study is the first to achieve the quantitative assessment of SGN numbers by directly injecting radiolabeled molecular probes into the cochlea and using antibody specificity to selectively label SGN membrane protein. The application of PET/SPECT, characterized by its high sensitivity and robust quantitative analysis capabilities, holds promise for the functional detection of the inner ear. Nevertheless, compared to traditional assessment techniques, nuclear medicine imaging approaches still face limitations such as higher radiation doses, elevated costs, and a certain degree of invasiveness.

Despite these challenges, nuclear molecular imaging has demonstrated significant clinical application potential in fields like oncology, enabling noninvasive evaluation of tumor metabolic activity or target protein expression, and serving as a critical tool for patient diagnosis and treatment prediction.^[^ ^43^ ^]^ For example, FDG‐PET predicts breast cancer patient response to HER2‐targeted therapy by quantifying glucose uptake, thereby informing precision chemotherapy selection.^[^ ^44^ ^]^ Similarly, Trop2‐targeted molecular imaging (e.g., ⁶⁸Ga/⁸⁹Zr‐labeled antibodies) allows visualization and quantification of Trop2 expression in tumors via PET/SPECT imaging, supporting cancer staging and treatment decisions.^[^ ^45^ ^]^ In neurodegenerative diseases, ¹⁸F‐labeled amyloid plaque tracers (e.g., ¹⁸F‐florbetapir) enable noninvasive detection of brain amyloid deposits using PET imaging, serving as a key basis for early AD diagnosis.^[^ ^46^ ^]^ Furthermore, Sugyo et al. used ^89^Zr‐labeled TfR‐targeting mAb TSP‐A01 to image a mouse pancreatic cancer model, showing that ^89^Zr‐labeled TSP‐A01 clearly visualized MiaPaCa‐2 tumors with high TfR expression.^[^ ^47^ ^]^ Similarly, immune‐PET imaging targets mesothelin (MSLN) overexpression, a prognostic marker in pancreatic cancer. ^89^Zr‐labeled MSLN‐targeting antibodies enabled clear visualization of tumor lesions and antibody distribution in pancreatic and ovarian cancer patients before ADC treatment.^[^ ^48^ ^]^ In the future, integrating auditory research with nuclear medicine holds significant potential. Combining radionuclide tracers with specific inner ear markers could enable in vivo detection of key protein expression levels and further reflect auditory function in the inner ear.

However, our study has several limitations that must be addressed before clinical application: 1) In terms of model selection, it should be noted that the present study focused on the clinically critical population of very severe sensorineural deafness due to defects in the *GJB2* gene. Therefore, the study employed Sox2CreER Cx26‐null mice as the SGN injury model. Additional animal models with different pathogenic mechanisms (e.g., ototoxicity model and aging model) could further validate this strategy from multiple dimensions and yield valuable insights. Notably, large animal models such as pigs are critical for evaluating the clinical translatability of this nuclear imaging approach. We plan to perform dedicated ¹⁷⁷Lu‐DOTA‐anti‐VGLUT1 imaging studies in pigs to comprehensively assess imaging performance, pharmacokinetics, and safety. 2) For the administration method, we employed RWM injection in mice to enable radionuclide diffusion through the scala tympani into Rosenthal's canal, thereby achieving targeted SGN labeling. Although effective, it exhibits limitations, notably potential cochlear damage, which highlights the need for noninvasive or minimally invasive alternatives. Thus, we are actively investigating the systemic administration. This approach is distinguished by its operational simplicity, high accessibility, and noninvasive nature. It involves directly injecting radiopharmaceuticals (e.g., ¹⁸F‐FDG, ⁶⁸Ga‐DOTATATE) into the bloodstream via intravenous injection, which has become the predominant method for PET/CT in clinical practice. However, in the inner ear, the presence of the BLB limits the penetration of large‐molecule drugs into the cochlea. To overcome this challenge, we aim to develop small‐molecule tracers (e.g., nanobodies), or probes attached to peptides that can be targeted to penetrate the BLB to enable radionuclide probes to cross the barrier and reach the cochlea.^[^ ^49^, ^50^ ^]^ This would facilitate noninvasive in vivo imaging and expedite the clinical translation of this technology. 3) No damage was observed in cochlear structures, including HCs and SCs of OC, in pig and mouse models after radionuclide administration. This suggests that the radionuclide probe does not cause short‐term damage to cochlear structures. However, long‐term monitoring of auditory function and structure is required to confirm its safety and absence of toxicity.

## 4 Conclusion

In conclusion, this study demonstrates that VGLUT1 is a promising marker for SGNs. The nuclear molecular probe, developed by coupling VGLUT1 with DOTA and radiolabeling it with ^177^Lu, enables in vivo imaging of the SGN and visualization of target antigen expression in the cochlea. This enables visual assessment of the relative SGN quantity in the cochlea. This strategy could predict postoperative outcomes in CI candidates and identify beneficiaries before surgery, offering valuable guidance for clinical decision‐making. Thus, this study highlights the application potential and translational value of nuclear molecular probes in auditory nerve research, pioneering a novel approach in neuroimaging within nuclear medicine.

## 5 Experimental Section

### Animal Models

Two animal models were utilized in this study. The transgenic mouse models Cx26^loxP/loxP^ and Sox2CreER were offered by Prof. Lin of Emory University and Prof. Chai of Southeast University. Tamoxifen‐inducible Cx26‐null mice were created by crossing the sexually mature Sox2CreER with the Cx26^loxP/loxP^ mice. Tail genomic DNA was used for genotyping of mice via polymerase chain reaction (PCR) amplification.^[^ ^23^ ^]^ The primers for genotyping were as follows:

Cx26 (F): 5′‐ACAGAAATGTGTTGGTGATGG‐3′,

Cx26 (R): 5′‐CTTTCCAATGCTGGTGGAGTG‐3′,

Sox2‐Cre (F): 5′‐AGCTAAACATGCTTCATCGTCGGTC‐3′,

Sox2‐Cre (R): 5′‐TATCCAGGTTACGGATATAGTTCATG‐3′.

Tamoxifen (T5648‐1G, Sigma‐Aldrich) (total dose of 1.5 mg/10 g body weight) was injected subcutaneously with a dose of at P0 and P1. The Sox2CreER Cx26‐null mice and their Cx26^loxP/loxP^ littermates served as the experimental and control groups, respectively. All mice were raised in the specific‐pathogen‐free Experimental Animal Centre of Huazhong University of Science and Technology.

The Bama miniature pig was used to establish the SGN injury model. Using an endoscopic trans‐tympanic membrane puncture, ouabain (Sigma, PHR1945, 10 mm) was administered through the RWM to destroy SGNs in the cochlea, while the other ear was untreated as the control. The pigs were housed in the stainless‐steel cage.

All animals were provided adequate breeding feed and high‐pressure filtered water and housed under a 12‐h light/dark cycle at a constant temperature of 21–23 °C. All experimental procedures were approved by the Committee on Animal Research of Tongji Medical College, Huazhong University of Science and Technology ([2021] IACUC Number: 3638).

### Auditory Brainstem Response in Mice

Hearing thresholds were measured by ABR at 1‐month. Mice were anesthetized with a compound anesthetic, ketamine hydrochloride (120 mg kg^−1^) mixed with chlorpromazine hydrochloride (20 mg kg^−1^). Measurements were made in a special quiet room and the mice were put on the 37 °C thermostatic electric blanket. The recording electrode and reference electrode were inserted under the skin of the skull or the tested ear, respectively. The speaker was positioned 5 cm away from the mouse's ear. The Tucker‐Davis Technology (TDT) RZ6 auditory workstation was employed to measure the hearing threshold. ABR responses were elicited and subsequently amplified (10 000 times), filtered (300‐3kHz passband), and averaged (512 responses). The sound stimulus started at an intensity of 90 dB and decreased stepwise to a subthreshold level in 5 dB steps. Six frequencies (8, 12, 16, 20, 24, 28, 32, and 40 kHz) were tested, and the SigGen32 software (Tucker–Davis Technologies) was used to amplify and record the ABR signals at different frequencies. The threshold was determined as the lowest SPL at which a reproducible ABR waveform could be detected. If no ABR wave was detected at maximum intensity stimulation, a nominal threshold of 90 dB was assigned.

### ABR in the Bama Miniature Pig

The anesthesia procedure for the Bama miniature pig was similar to that used for mice. Electrodes were inserted subcutaneously at the vertex and behind the pinna on both sides. During ABR tests, click and different sound intensities (10–90 dB) were generated and delivered using a TDT RZ6 auditory workstation. The detailed procedure followed previously described protocols.

### Protein Extraction and Western Blotting

The Sox2CreER Cx26‐null mice and their Cx26^loxP/loxP^ littermates were anesthetized and sacrificed at P7. There were three biological replicates for each of the experimental and control groups. Total protein was obtained from the cochleae by means of RIPA lysis buffer (P0013B, Beyotime). Samples with equivalent amounts of protein were segregated through 12.5% sodium dodecyl sulfate‐polyacrylamide gel electrophoresis (SDS‐PAGE) and subsequently transferred onto polyvinylidene difluoride (PVDF) membranes. After blocking in TBST (Tris‐buffered saline with 0.1% Tween‐20) containing 5% milk for 1 h, the Cx26 and β‐actin proteins were identified using anti‐Cx26 antibody (710500, Invitrogen) or anti‐β‐actin antibody (GB15003‐100, Servicebio), respectively. The PVDF membranes were then incubated with horseradish peroxidase‐conjugated secondary antibodies. The protein bands were visualized using an ECL reaction kit, and band intensities were semi‐quantified.

### RNA Preparation and Real‐Time Quantitative Polymerase Chain Reaction (RT‑qPCR)

Total RNA was extracted from SGNs and the other components of cochleae with Trizol Reagent (RK30129, ABclonal) and then reverse transcribed by using PrimeScript RT reagent kit with gDNA eraser (RR047A, Takara). RT‐qPCR was performed using SYBR Premix Ex Taq (RR420A, TaKaRa Bio) with GAPDH as the housekeeping gene. The results were analyzed using the comparative cycle threshold 2^−ΔΔCT^ method. The following primers were used for RT‐qPCR:

GAPDH (F): 5′‐GAAGGTCGGTGTGAACGGAT‐3′

GAPDH (R): 5′‐CTCGCTCCTGGAAGATGGTG‐3′

VGLUT1 (F): 5′‐GGACATCGCCCCTCGATAT‐3′

VGLUT1 (R): 5′‐ GTGTGCCCACGCCATTG‐3′

### Cochlear tissue preparation and immunofluorescent labeling

The cochlear specimens from mice and pig were acquired after euthanasia. A small incision was made at the apical tips to ensure proper fluid exchange during immersion fixation with 4% polyformaldehyde for 2 h at room temperature and then decalcified in ethylenediaminetetraacetic acid (EDTA) for 48 h. For frozen sections, the decalcified cochlear tissues were placed in a gradient sucrose solution for dehydration by immersion in 10%, 20%, and 30% sucrose solutions for 1 h each and then embedded in optimal cutting temperature compound overnight at 4 °C. These samples were sectioned at 10 µm for subsequent procedures. For flattened cochlear preparations, cochleae were dissected into three turns (apical, middle, and basal) under the microscope. The sections or flattened cochlear preparations were blocked in 5% bovine serum albumin for 1 h, then incubated overnight at 4 °C with anti‐Cx26 antibody (710500, Invitrogen), anti‐VGLUT1 monoclonal antibody (ab227805, Abcam), anti‐KCNJ10 antibody (Alomone Labs Cat#APC‐035, RRID: AB_2040120), anti‐βIII‐tubulin antibody (Cat# 4466, Cell Signaling). Then the samples were washed with PBS and incubated with secondary antibody for 2 h at room temperature. The samples were counterstained with DAPI (C1005, Beyotime Biotechnology) and phalloidin (0.05 mg mL^−1^, P5282, Sigma–Aldrich) to label the nucleus and F‐actin and then observed with laser‐scanning confocal microscope (Nikon).

### SGN Counting

SGNs were quantified in frozen sections which were observed using the laser‐scanning confocal microscope, similar to. Serial sections were obtained. The 4–6 sections were stained every 64 µm from each turn of the cochlea, and only sections where the ganglion structure was intact, as assessed through bright‐field images of these structures, were used in quantification. SGNs were immunolabeled with the anti‐KCNJ10 antibody (Alomone Labs Cat#APC‐035, RRID: AB_2040120) and the anti‐βIII‐tubulin antibody (Cat# 4466, Cell Signaling). NIH ImageJ software was used to count the total number of SGNs. Photoshop 2024 was used to generate masks to measure the area of Rosenthal's canal. The area of these masks was measured using ImageJ. The 6–10 cochleae per condition were processed for SGN quantification. The number of surviving SGNs in the cochlear apical, middle, and basal turns was divided by the area of Rosenthal's canal. All images of SGNs for each condition were blinded, randomized, and counted by a different individual. The SGN density per unit area (µm^2^) was calculated. Finally, SGN density at each turn was averaged for each cochlea, and these biological replicates were averaged. For VGLUT1 quantification, the number of VGLUT1 positive cells per unit area in the apical, middle, and basal turns of the cochlea was determined as mentioned above. Four cochleae per condition were processed for VGLUT1 positive cell quantification. Statistical significance was determined by ANOVA.

### RWM Injection in Mice

Adult mice at 3 m were anesthetized by intraperitoneal injection of a compound anesthetic, ketamine hydrochloride (120 mg/kg) mixed with chlorpromazine hydrochloride (20 mg kg^−1^). The anesthetic depth was assessed by performing noxious stimulation tests, such as pinching the paws and the tail. After shaving the hair surrounding the left ear and neck area of the mouse, the mouse was placed in the lateral position. The postauricular area was disinfected following the principles, and a small incision ≈0.5 cm was created behind the ear. Under the microscope, fat and muscle were dissected until the tympanic cavity was uncovered. Subsequently, a hole was created in the upper right of the tympanic cavity using the beveled tip of a needle. At that point, the round window above the stapedial artery was exposed. After aspirating the imaging agent with a microinjector, it was inserted into the RWM, and the “breakthrough sensation” of the membrane rupture was felt. Subsequently, the microinjector was inserted from the round window into the scala tympani to approach the Rosenthal s canal as closely as possible. At this point, a total of 2 µL of radioactive tracer was gradually injected into the cochlea. Finally, a small piece of muscle was employed to fill the RWM, and the exposed wound was sutured layer by layer. The mice were placed on a 37 °C constant‐temperature electric blanket for resuscitation.

### Preparation and Radiolabeling of ^177^Lu‐DOTA‐Anti‐VGLUT1

VGLUT1 monoclonal antibody (ab227805, Abcam) was conjugated with DOTA at a molar ratio of 1: 50 (mAb: chelator). The mixture was incubated in the dark at room temperature for 2 h with gentle shaking to obtain the DOTA‐anti‐VGLUT1 antibody complex. Subsequently, the complex was purified using a PD10 column with PBS, and the concentration of DOTA‐anti‐VGLUT1 was quantified by UV absorbance using an ELISA reader. For ^177^Lu labeling, a labeling buffer was prepared by mixing 6 mL of 0.05 m HCl with 390 µL of 1.0 m sodium acetate to achieve a pH of ≈4.0. A neutralization buffer (0.1 m Na_2_CO_3_) was also prepared. To initiate the labeling reaction, 0.5 mL of the labeling buffer was added to the pre‐configured DOTA‐anti‐VGLUT1 solution in reaction vial 1. Next, 0.5 mL of labeling buffer was added to the ^177^Lu vial (100 m Ci/vial), and 0.6 mL of the radionuclide solution was drawn and added to reaction vial 1. This step was repeated twice to ensure a complete transfer of ^177^Lu. The reaction vial was then incubated at 95 °C for 15 min. Radio‐TLC and Radio‐HPLC analyses confirmed a radiochemical purity of >95%. Finally, the pH was adjusted to 7.0 by adding 0.15 mL of 0.1 m Na_2_CO_3_ per 1 mL of reaction solution, followed by filtration through a 0.22 µm microporous membrane. The labeled product was diluted with 2.0 mL of physiological saline and stored at 4 °C for future use. The entire process adhered to standard operating procedures for radiolabeling.

### PET Imaging of ^11^C‐CFT in the Bama Miniature Pig

For the Bama miniature pig, the dopamine transporter imaging agent ^11^C‐CFT (5 mCi) was intravenously pushed into the left limbal vein after anesthetization. Scanning was conducted 10 min later. The CT scan was completed with a tube voltage of 120 kV, a tube current of 80 mA, a slice thickness of 3.75 mm, and an interslice distance of 3.27 mm. For the planar scan of the ET brain, one‐bed position was used, with acquisitions conducted every 10 min for 15 min each, totaling four times. The PET scan was performed in 3D mode, and attenuation correction was based on the CT data and reconstructed using the OSEM method.

### PET Imaging of ^11^C‐CFT and SPECT Imaging of ^177^Lu‐DOTA‐Anti‐VGLUT1 in the Mice

Nuclear imaging was performed using an Inveon PET/SPECT scanner (Siemens) after RWM injecting 2 µL of ^11^C‐CFT (≈20.3 MBq) and ^177^Lu‐DOTA‐anti‐VGLUT1 (≈12.2 MBq). At the 1 and 12 h postinjection (p.i.) time points, mice were anesthetized and scanned in a prone position. The acquisition time for each scan was ≈40 min. The 20–30 million coincidence events were obtained for each mouse. After attenuation corrected reconstruction, quantitative radioactivity results were acquired by drawing regions of interest (ROI) at the cochleae and analyzed using an Inveon Research Workspace (Siemens). Due to the homogeneity of the cochlea's weight, tissue uptake values are presented as the percent injected dose (%ID). This procedure was repeated three times. After the final PET/SPECT scans at 12 h p.i., the bilateral cochleae were harvested for ex vivo nuclear imaging. Ex vivo biodistribution studies were performed to quantify the ^177^Lu‐DOTA‐anti‐VGLUT1 uptake in the cochleae.

### Data Analysis of Single‐Cell Transcriptomic Data

Single‐cell transcriptomic data GSE202920 was obtained for the cochlear tissue samples of C57/B6 mice at P28 from the Gene Expression Omnibus (GEO) database (https://www.ncbi.nlm.nih.gov/geo/).^[^ ^51^ ^]^ Two samples each consisting of 14 cochlear tissues from this dataset were utilized for further analysis. To ensure cell quality, genes expressed in fewer than three cells in the sample are filtered out, along with cells having gene expression less than 200. Doublets were removed using Scrublet. Cells with >22.62% mitochondrial genes and >46.13% Ribosomal genes were also excluded from the analysis. The data matrix is normalized using the “scanpy.pp.normalize_total” function from Scanpy. Subsequently, dimensionality reduction and unsupervised clustering are carried out based on the workflow in Scanpy. The top 4000 variable genes are calculated by means of the “scanpy.pp.highly_variable_genes” function. The “scanpy.pp.regress_out” function was employed to regress out the influences of the total count of each cell, the percentage of expressed mitochondrial genes, and the percentage of ribosomal genes. Moreover, “scanpy.pp.scale”was utilized with the parameter “max_value = 10” to scale each gene to unit variance. After data preprocessing, the dimensionality of the data is reduced through conducting principal component analysis (PCA). To eliminate batch effects from different datasets, batch correction is performed using the “sc.external.pp.harmony_integrate” function with the parameter “n_pcs = 47”. Furthermore, the dimensionality of the merged dataset is decreased using the UMAP implemented by the “scanpy.tl.umap” function. Then, the cells in the neighborhood graph are clustered using the Leiden clustering method. To visualize and investigate the datasets, a nonlinear dimensional‐reduction technique, t‐distributed Stochastic Neighbor Embedding (tSNE) was implemented using the function “RunTSNE”. In addition, the VGLUT1 expression in various cells was visualized using ballon plots created with the ggplot2 package.

### Data Analysis

All the images were linearly processed with ImageJ software (Fiji, Inc.). All data were presented as mean ± SEM and analyzed using GraphPad Prism 9.0 (GraphPad Software Inc., La Jolla, CA, USA). The statistical significance was determined using paired or unpaired two‐tailed Student's *t* tests when comparing two groups. When performing the unpaired *t*‐test, the F‐test analysis revealed homogeneity of variance among the groups (*p* > 0.05). Differences among multiple groups with one or two variables were determined using one‐way or two‐way ANOVA respectively. *p *< 0.05 was considered statistically significant.

## Conflict of Interest

The authors declare no conflict of interest.

## Author Contributions

C.K., X.W., and J.Y. contributed equally to this work. Y.S., D.J., and X.L. conceived and designed the experiments. Material preparation, data collection, and analysis were performed by C.K., J.Y., L.W., C.Z. Investigation, data curation, and software supervision were conducted by G.Y., K.X., W.H., and H.W. The first draft of the manuscript was written by C.K. and X.W. All authors commented on previous versions of the manuscript. All authors read and approved the final manuscript.

## Data Availability

The data that support the findings of this study are available from the corresponding author upon reasonable request.
